# Coach-Supported Versus Self-guided Digital Training Course for a Problem-solving Psychological Intervention for Nonspecialists: Protocol for a Pre-Post Nested Randomized Controlled Trial

**DOI:** 10.2196/41981

**Published:** 2023-06-13

**Authors:** Sonal Mathur, Helen A Weiss, Melissa Neuman, Andy P Field, Baptiste Leurent, Tejaswi Shetty, James E J, Pooja Nair, Rhea Mathews, Kanika Malik, Daniel Michelson, Vikram Patel

**Affiliations:** 1 Sangath New Delhi India; 2 Medical Research Council International Statistics and Epidemiology Group Faculty of Epidemiology and Population Health London School of Hygiene and Tropical Medicine London United Kingdom; 3 School of Psychology University of Sussex Brighton United Kingdom; 4 Department of Statistical Science University College London London United Kingdom; 5 Jindal School of Psychology and Counselling O.P. Jindal Global University Sonipat India; 6 Department of Child and Adolescent Psychiatry Institute of Psychiatry, Psychology and Neuroscience King's College London London United Kingdom; 7 Department of Global Health and Social Medicine Harvard Medical School Boston, MA United States

**Keywords:** adolescent mental health, capacity building, digital training, India, problem-solving intervention, randomized controlled trial

## Abstract

**Background:**

Psychosocial interventions delivered by nonspecialists can be effective at reducing common adolescent mental health problems in low-resource settings. However, there is a lack of evidence on resource-efficient methods for building capacity to deliver these interventions.

**Objective:**

The objective of this study is to evaluate the effects of a digital training (DT) course, delivered in a self-guided format or with coaching, on nonspecialists’ competency to deliver a problem-solving intervention intended for adolescents with common mental health problems in India.

**Methods:**

We will conduct a pre-post study with a nested parallel, 2-arm, individually randomized controlled trial. The study aims to recruit 262 participants, randomized 1:1 to receive either a self-guided DT course or a DT course with weekly individualized coaching provided remotely by telephone. In both arms, the DT will be accessed over 4 to 6 weeks. Participants will be nonspecialists (ie, without prior practice-based training in psychological therapies) recruited from among university students and affiliates of nongovernmental organizations in Delhi and Mumbai, India.

**Results:**

Outcomes will be assessed at baseline and 6 weeks post randomization using a knowledge-based competency measure that incorporates a multiple-choice quiz format. The primary hypothesis is that self-guided DT will lead to increased competency scores among novices with no prior experience of delivering psychotherapies. The secondary hypothesis is that digital training with coaching will have an incremental effect on competency scores compared with DT alone. The first participant was enrolled on April 4, 2022.

**Conclusions:**

The study will address an evidence gap on the effectiveness of training methods for nonspecialist providers of adolescent mental health interventions in low-resource settings. The findings from this study will be used to support wider efforts to scale up evidence-based mental health interventions for young people.

**Trial Registration:**

ClinicalTrials.gov NCT05290142; https://clinicaltrials.gov/ct2/show/NCT05290142

**International Registered Report Identifier (IRRID):**

DERR1-10.2196/41981

## Introduction

Task-sharing is increasingly used to address unmet mental health needs in low-resource settings. Task-sharing approaches deploy trained community and voluntary sector workers in service delivery roles that are traditionally held by specialized professionals [1]. Trials have demonstrated the effectiveness of resource-efficient “low-intensity” psychological interventions delivered by nonspecialists [2,3], but there is a lack of evidence on how to make these solutions work sustainably.

Training capacity has been a major supply-side barrier, with traditional face-to-face methods requiring a critical mass of expert trainers and physical infrastructure [4]. The growing use of web-based educational materials, which accelerated during the COVID-19 pandemic, presents significant opportunities for building workforce capacity at scale [5,6]. The supporting evidence from the global mental health literature suggests benefits of digital training (DT) in terms of time, efficiency, flexibility, and reduced economic costs [7]. However, differences in training approaches and evaluation measures limit comparability of findings across studies. Moreover, key engagement challenges identified for digital learning from the wider pedagogical literature leading to relatively high rates of dropout [8-10] have not been systematically addressed or studied in relation to nonspecialist providers of mental health care.

This study addresses the lack of evidence on workforce development strategies for scaling up evidence-based adolescent mental health interventions in low-resource settings. The aim is to evaluate 2 versions of DT for nonspecialists, recruited from a variety of contexts in India, and compare their effects on knowledge-based competency needed to deliver a brief evidence-based problem-solving intervention for common adolescent mental health problems. The effects of the problem-solving intervention on adolescent mental health have been previously evaluated in a randomized controlled trial in Indian secondary schools where lay counselors were trained using face-to-face methods [3]. In this study, we are interested in assessing the effects of DT on competency in a nonspecialist workforce that could deliver the problem-solving intervention more widely.

We are particularly interested in the potential incremental effects of providing personalized coaching for learners on a DT course. A recent pilot study involving nonspecialist primary care workers in Madhya Pradesh, India, demonstrated that the addition of remote telephone coaching improved training completion rates for a digital program [11], for training frontline community health workers on a brief psychological treatment for adults with depression [12]. We wished to test whether a similar coaching approach may enhance engagement with, and competencies attained, with a DT package related to an adolescent mental health intervention.

Our specific objectives are to (1) evaluate the effects of DT on knowledge-based competency of trained nonspecialists to deliver an evidence-based problem-solving intervention for common adolescent mental health problems; (2) evaluate the incremental effect of digital training with coaching (DT-C) in comparison with self-guided DT on nonspecialists’ competency to deliver the problem-solving intervention; and (3) investigate the implementation of the training intervention in both arms.

## Methods

### Design 

This protocol adheres to the Standard Protocol Items: Recommendations for Interventional Trials guidelines [13,14]. A parallel, 2-arm, individually randomized controlled trial design will be nested within a pre-post intervention study. Outcomes will be assessed at baseline and 6 weeks post randomization. 

### Setting 

This study will be conducted in partnership with 4 universities (2 coeducational colleges in the National Capital Territory of Delhi, 1 coeducational college in Bangalore, Karnataka, and 1 girls-only college in Mumbai, Maharashtra), and 5 nongovernmental organizations (NGOs) working in the fields of adolescent health and education. The sampling frame comprises approximately 2500 university students and 2500 staff and volunteers associated with NGOs.

### Participants

Participants will be sampled from two broad groups: (1) university students currently enrolled in a bachelor’s-level degree program in psychology, education, or allied fields; and (2) NGO employees who are working as teachers, social workers, or mental health advocates. Eligible participants in both groups will be aged 18 years or older; be fluent in written and spoken Hindi or English; and have regular access to a web-enabled smartphone or computer that can be used to participate in the digital course. Potential participants will be excluded from the study if they have prior practice-based training in psychological therapies. 

### Interventions

#### Self-guided DT Arm: Digital Content

The DT course comprises 16 modules organized into 2 sections: nonspecific counseling skills and skills that are specific to problem-solving counseling. The course content is based on an existing manual for the Premium for Adolescents (PRIDE) problem-solving intervention [2], which was designed in India as a first-line treatment within a transdiagnostic stepped care architecture for adolescents with elevated mental health concerns of varying types. A previous randomized controlled trial in India showed that the problem-solving intervention effectively reduces adolescents’ self-rated psychosocial problems and mental health symptoms when implemented by relatively inexperienced lay counselors in four or five 20- to 30-minute sessions held across 3 weeks [3,15]. The manual was translated into a DT course in 5 steps as described in Textbox 1, following a structured process established in previous research [16].

**Intervention development process.**

**Create curriculum outline**

- A course outline was developed to ensure alignment between the learning objectives and the core content of the Premium for Adolescents (PRIDE) problem-solving intervention manual
- Three members of the current study team (SM, TS, and RM) drafted the course blueprint, which was reviewed by 3 of the original PRIDE manual developers (DM, CF, and VP) to ensure that all key competencies were represented

**Develop training content**

- Written content from the PRIDE manual was adapted into multimodal content including videos, text, and PowerPoint for the training course
- Draft materials were reviewed again by the original PRIDE manual developers to ensure faithful translation of concepts and procedures

**Digitize training content**

- Materials were produced in digital formats that could be accessed from both a smartphone app and website
- These included video-based lectures, role plays, animated summary slide shows, and quizzes
- Materials were checked for accuracy and visual appeal by members of the study team

**Adding content to a learning management system (LMS)**

- Digital content was published on an open-source web-based LMS
- The platform included learner performance tracking and metrics, a customizable interface available in multiple languages, and a user-friendly dashboard

**Conduct user testing**

- User testing was carried out in a group format with 20 undergraduate psychology students
- On the basis of feedback received, the app was further optimized for viewing video content, a message repository was prepared to send automated notifications to motivate learners
- The numbering of videos was made simpler, the glossary was expanded, and a recruitment video was prepared

The course will be hosted on a learning management system (LMS) hosted by Sangath, the implementing organization in India, which can be accessed through a smartphone app and website in either Hindi or English, based on the user’s preference. The content will be delivered through a variety of instructional methods including video-based lectures, role-play videos, animated summary slide shows, quizzes, and brief readings adapted from the source problem-solving manual. The training requires approximately 20 hours to complete, accounting for the time needed to engage with the various content areas. This can be spaced out over 4 weeks, with 4 modules being unlocked every week to facilitate sequential learning. Participants who are unable to complete the course within 4 weeks will be offered an extension for up to 2 weeks during the trial, after which their access to the course will be locked for the duration of endline assessment.

Automated support features include weekly emails and text notifications on the LMS platform that serve as reminders and motivators to engage with the digital content. In addition, participants will be able to send technical queries (eg, related to accessing and navigating the digital platform) to a dedicated WhatsApp number. This account will be monitored during regular working hours by a research assistant who will provide support accordingly, while declining to answer any course content-related queries.

#### DT-C Arm

##### Coaching

In addition to the DT program, participants in the DT-C arm will receive personalized remote coaching for the duration of the course. The main functions of the coach are to (1) review core concepts from the course content, (2) respond to specific content-related queries, and (3) reinforce progress toward completing the course in the allocated time frame. There will be 4 coaches, each of whom will be bilingual and possess an undergraduate degree. Coaches will have completed training in the PRIDE problem-solving intervention, as well as receiving 5 days of office-based training in the coaching protocol that was designed specifically for the current study. The coaching protocol (see the outline in Table 1) has been adapted from 2 existing psychotherapy coaching protocols intended for nonspecialists [11,17].

**Table 1:** Outline of the coaching protocol.

Content sections (activities done by the coach)	Description
Build rapport	Engage the learner and create a collaborative space for coaching
Orient learner	Introduce learner to the course and coaching and set expectations
Plan for course completion	Discuss timetable over the intended 4-week course duration, including time required for each module and schedule for weekly coaching calls
Review progress	Assess progress on a weekly basis and troubleshoot challenges to completion
Resolve queries	Answer any queries from the learner about course content
Provide motivation	Encourage the learner by verbally reinforcing progress
Set learning goals	Collaboratively set learning goals for week ahead
Wrapping up	Recap material and discuss potential for skills practice and application

Coaching will be delivered through voice-based telephone calls and SMS text messages. The coach will call participants once per week over the 4-week duration, which may be extended by up to 2 weeks. Each coaching call is intended to last for approximately 30 minutes and follow a structured agenda. Prior to the call, the coach will review key performance metrics (modules completed and scores on formative quizzes) collected from the LMS dashboard. During each call, the coach will check if the participant has experienced progress-related challenges, clarify any difficulties with course concepts, reinforce continued participation, and collaboratively set learning goals for the week ahead. Participants will also have the option to ask queries via SMS text messages to their coach between calls. Depending upon the nature of the query, a coach may respond over text or address the query during the next scheduled phone-based coaching session. Additionally, coaches will send a reminder message to learners 1 day prior to scheduled coaching calls. 

Throughout the trial, coaches will participate in weekly peer group supervision meetings lasting 2 to 3 hours. Group supervision will include quality ratings of audio-recorded coaching sessions and trouble-shooting around logistical/technical issues, which will be moderated by a supervising masters- or doctoral-level clinical or counseling psychologist. The coaches will be able to make ad hoc calls to a supervising psychologist in case of any pressing concerns outside the group supervision sessions.

Coaches will not supervise learners in the applied use of the problem-solving intervention for treating adolescent mental health problems. The study recruitment materials will emphasize that a suitably experienced and qualified clinician should be engaged in supervising any such clinical activity with appropriate quality assurance and safeguarding arrangements in place. An outline of the coaching components is given in Table 1.

##### Procedure

The study schedule is summarized in Table 2 and set out in further detail below.

**Table 2:** Schedule for enrolment, interventions, and assessments.

Time point		Baseline (week 0)	Intervention (weeks 0-24, with a 2-week allowance)	Follow-up (week 6, with a 2-week allowance)
**Procedures**				
	Eligibility screening	✓	N/A^a^	N/A
	Informed consent	✓	N/A	N/A
	Baseline assessment and allocation	✓	N/A	N/A
	Intervention		✓	
	Follow-up assessment			✓
**Data collection**				
	Demographic information	✓	N/A	N/A
	KOPS^b^	✓	N/A	✓
	MUSIC^c^			✓
	LMS^d^ process data		✓	
	Coaching process data		✓	

^a^N/A: not applicable.

^b^KOPS: knowledge of problem solving scale.

^c^MUSIC: eMpowerment, usefulness, success, interest, and caring scale.

^d^LMS: learning management system.

#### Recruitment and Consent

Participants will be recruited through sensitization webinars that will raise awareness about the study and opportunities to participate in the DT course. The webinars will be coordinated with the 9 partner organizations (NGOs and universities) using existing communication channels such as email lists and WhatsApp groups. Sessions will be conducted throughout the recruitment period to maintain a consistent flow of referrals until our target sample is achieved. Webinars will be facilitated by a member of the intervention team using a PowerPoint presentation, video demonstration, and Q&A. A telephone number and REDCap platform URL [18] will be provided to individuals who wish to begin the enrollment process. Where possible, physical posters, with information about the study and associated training opportunities, will also be displayed in preselected locations on the premises of the collaborating organizations.

Following sensitization activities, interested individuals will be able to initiate a referral by directly visiting the study website, or by contacting a dedicated centralized number (via call or SMS text message) to request further information and leave their preferred contact details. A research assistant will respond by emailing and, if required, by texting a link to the study website (hosted on the REDCap platform). When accessing the REDCap platform, they will be able to read information about the study before completing a series of eligibility questions on the web. The questions will ask about age, current occupation, device access, preferred language for training, and prior training or other experience in delivering psychological therapies. Individuals who require assistance to complete the eligibility form will be able to contact the study helpline and speak to a research assistant. Details of the helpline contact will be shared through the recruitment posters and messages shared via WhatsApp and email. If an individual leaves the eligibility screening form incomplete for 2 days, a research assistant will contact the participant to identify and clarify any difficulties they are facing. If the participant is ineligible for the study, then they will not be followed up by the research team and instead redirected to the Sangath website to view other information about alternative courses. Once eligibility is confirmed, a prospective participant will be emailed a weblink to the study information sheet and consent form. Each consenting participant will be asked to complete a demographic proforma (see “measures” below).

#### Baseline Assessment

Consenting individuals will be directed to complete the baseline assessment measure (see “measures” below) via a weblink contained within a further email. Each participant will also have the option to contact the study helpline if they face technical difficulties during the assessment. If the baseline assessment has not been completed within 2 days after enrollment, a research assistant will make contact by telephone to remind the participant. If needed, research assistants will make up to 4 more attempts to contact the individual over the subsequent 2 weeks.

#### Randomization and Allocation Concealment

Participants will be randomized in a 1:1 allocation ratio to 1 of the 2 trial arms. Randomization will be stratified by the type of organization, using randomly sized blocks of 4 or 6. The randomization sequence for each stratum will be generated by an independent statistician and deployed on REDCap by the data manager on site. The trial principal investigator, trial coordinator, trial statisticians, and members of the Trial Steering Committee will remain blinded to allocation arms throughout the trial and until the final analysis is complete. It will not be possible to blind the participants or coaches to the allocation because of the nature of the intervention. The data manager will also not be blinded. A separate Data Monitoring Committee was not established, since this is not clinical trial and there is no risk to the safety or participants.

An onboarding email will be sent to the participant informing them of their allocation status within 24 hours of randomization. This email will also contain details of the course and study structure, login details to access the DT platform, and a link to a tutorial video explaining participants how to access and navigate the training platform. Participants in the DT-C arm will additionally receive an introductory onboarding call from their coach to orient them toward coaching within 24 hours of randomization. A daily check will be performed by the data manager to evaluate if allocations are consistent with the allocation code.

#### Follow-Up Assessment

A link to the follow-up assessment materials will be shared with participants using their registered email address at 42 days (ie, 6 weeks) post randomization. The course has been structured in such a way that each week, 4 modules are unlocked for the learners. This would ensure that the learners can comfortably complete the course within 4 weeks. However, we have given the provision of additional 2 weeks for the learners to complete the course. Research assistants will make up to 4 more attempts to contact the participant over the subsequent 2 weeks or until the follow-up assessment is completed on the study website. Participants will be given a maximum allowance of 2 weeks to complete the follow-up assessment.

### Measures

#### Participant Characteristics

Sociodemographic data will be collected at baseline on age, gender, education level, occupation, years of experience (if employed), year and course of study (if currently a student), and preferred language for receiving the course (English or Hindi).

#### Primary Outcome

Self-completed outcome data will be collected remotely through the REDCap platform at baseline and 6 weeks after randomization into the study. The primary outcome will be a knowledge-based competency measure, the knowledge of problem-solving (KOPS) scale. This was developed specifically for the current study and contains 17 multiple-choice questions organized around 5 session vignettes. The measure produces a score ranging from 0 to 17, where higher scores indicate higher levels of competency. The vignette-based format was informed by previous research on competency assessments for nonspecialists [19-21]. Twelve questions assess knowledge of specific problem-solving competencies (eg, problem identification) and 5 questions assess nonspecific competencies of relevance to psychotherapies generally (eg, rapport building). To enhance external validity, question domains were cross-referenced with problem-solving items from an established CBT competency framework implemented widely in the United Kingdom [22] and nonspecific counseling items from 2 existing competency measures used in previous studies with nonspecialist providers in South Asia [23-25]. The wording of questions and details of the case vignettes were refined through piloting with a reference sample that included both experienced counselors and novices. The process of developing the KOPS measure is described in detail elsewhere (S Mathur et al., unpublished data).

Parallel forms of the case vignette and quiz will be used at baseline and end line assessments (ie, respondents will be assessed on the same domains but with different cases and alternative question formulations at each time point). In each form, participants will be presented with a written case description followed by 5 session vignettes in a logical sequence. The participant will be required to respond sequentially to a set of multiple-choice questions after each of these vignettes (3 to 4 questions per set). The sequencing of the 2 forms will be determined at random. Participants will have up to 90 minutes to complete the quiz once they begin answering, after which the assessment link will expire. The remaining time available will be visible as a countdown clock on the assessment screen.

#### Secondary Outcomes

User satisfaction data will be obtained from participants in both trial arms using the MUSIC (eMpowerment, Usefulness, Success, Interest, and Caring) questionnaire. We will use a version of the MUSIC scale that has been adapted in previous DT trials in India [11,26]. The scale will include 26 items, each rated on a 6-point scale, covering domains of feasibility, acceptability, adoption, and appropriateness. The questionnaire provides a score on each of the 5 scales (empowerment, usefulness, success, interest, and caring), each based on 4 to 6 items. Two supplementary free-text items will be used to obtain written qualitative feedback from participants about what they enjoyed the most in the course, as well as suggestions for improvement.

#### Process Evaluation

Data on enrolment, randomization, and completion of assessment procedures will be obtained from the REDCap platform. This will include numbers and proportions of study referrals that are assessed, eligible, consented, and randomized (by host organization, age, and gender); and reasons for missed assessments, ineligibility, declined consent, and missed randomization. Session record forms and automated data captured from the LMS will be used to assess frequency of log-ins, number of modules completed, and time spent logged into the LMS. Additional process variables in the DT-C arm will include number and duration of attended coaching sessions (and number of missed coaching sessions and reasons for nonattendance). Fidelity of coaching will be assessed using independent ratings of audio-recorded coaching calls (based on 10% of recorded calls). Coaches will also document the frequency and duration of calls (and reasons for missed calls), as well as frequency of SMS text messages received and sent in response.

#### Study Size

For the primary hypothesis that the DT intervention improves competency score, a sample size of 262 participants will provide 80% power to detect an effect size of 0.19 (ie, a standardized mean difference [SMD] of post vs pretraining scores), assuming 20% loss-to-follow-up, and a 5% 2-sided significance level. This indicative effect size (SMD=0.19) was informed by a systematic review and meta-analysis of web-based learning evaluations which compared analogous learning conditions [27]. For the second hypothesis that the DT-C intervention is superior to the DT intervention, this sample size will provide 80% power to detect an effect size (standardized mean difference) of 0.39 between the DT and DT-C arms with a 1:1 allocation ratio between randomized arms. With the 20% loss-to-follow-up, we anticipate that 210 (80%) participants will complete follow-up and contribute to the endline analysis (105 per arm).

### Data Security and Management

The principal investigator (VP) will act as custodian of the data in accordance with regulations of the research sponsor (Harvard Medical School) and funder (Wellcome Trust, United Kingdom). Each participant will be assigned a unique ID and password to access both the DT course and secure REDCap platform for outcome assessments. Data will be stored on a secure and backed-up server operated by a trusted third party (Sangath) in a managed environment that protects against data loss and corruption, unauthorized access, and modification. To mitigate the risk of identifying participants from the data collected in the study, we will not record direct identifiers on study data. Data in its original form will be retained on the server and will be accessible only to core research team members who are directly involved in the study.

Participant information sheets (and instructions for completing the outcome measures) will clearly explain that scores will remain confidential and will not be shared with university authorities, employers, or any other third parties. Participants will be informed through the information sheet that they can withdraw from the study at any time and their data will be removed up to the point when the primary analysis is completed. Anonymized participant data and a data dictionary will be made available 12 months after study completion. Data will be shared after approval by the PRIDE principal investigator, following a reasonable submitted request.

### Statistical Analysis 

#### General Principles

Except otherwise specified, analysis will be conducted according to the arm participants were randomized to, regardless of intervention effectively received (“intention to treat” principle), CIs will be reported at the 95% level, and significance considered at a 2-sided 5% α.

#### Descriptive Statistics

**Figure 1:**
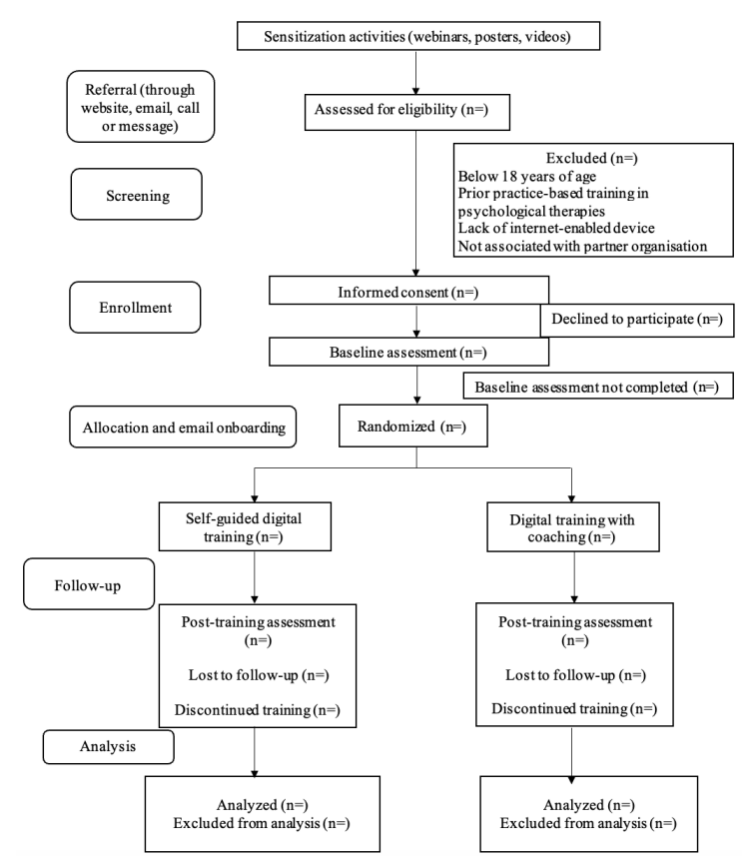
Consolidated standards of reporting trials (CONSORT) flow diagram.

We will describe participants’ baseline characteristics overall and by arm. A CONSORT flow diagram (Figure 1) will be used to summarize trial processes. Adherence to the intervention will be reported descriptively, including the number of modules and number of coaching calls completed. 

#### Psychometric Analysis

We will use the baseline data of outcome scores obtained from all participants to establish the psychometric properties of the multiple-choice KOPS competency measure. Prior to unblinding the trial data set, a 2-parameter Rasch model will be fitted to estimate the item characteristic curves, discrimination, and difficulty parameter for each item on both forms of the competency measure. In addition, the test information function will be estimated to look at the overall characteristics of the forms. Based on this analysis, items may be removed, and the primary outcome analysis will be conducted using retained items. Items that have poor discrimination or are at the very extremes of difficulty may be removed in the interests of a test information function that suggests that the scale as a whole provides information across a range of abilities. If any items are removed, a sensitivity analysis will be conducted in which the original 17-item version is used as the outcome for the model that tests the main trial hypothesis.

#### Missing Data

We will compare baseline characteristics of study completers versus noncompleters. The main analyses (hypotheses 1 and 2) will use multiple imputation under the missing at random assumption, by chained equations, and including the main predictors of missing data in the imputation model. Sensitivity analysis will include complete case analysis, adjusting for factors associated with missing data, and under missing not-at-random assumptions.

#### Analysis of Pre-Post Differences

The analysis of pre-post differences will be conducted by doing a linear regression of the change in competency score between baseline and 6 weeks. The null hypothesis will be that there is no change in score.

#### Randomized Controlled Trial Analysis

The second hypothesis will be analyzed by a linear regression of the change in competency score between baseline and 6 weeks, testing for a difference between the 2 arms. The analysis will be adjusted for strata, and baseline competency score.

#### Analysis of Effect Modification

To assess on whom the DT and additional coaching may work best, we will assess effect-modification on the primary and secondary outcomes by *a priori* defined modifier (age, gender, language, and type of organization [NGO vs university]), by including these factors as predictors (pre-post analysis), or as interaction terms (trial analysis) testing for heterogeneity of treatment effects in the regression models specified above.

#### Secondary Outcomes Analysis

The effect of coaching on the secondary outcomes (MUSIC 5 subscales and DT course completion) will be conducted similarly to the trial analysis model described above, but without adjusting for baseline score. The MUSIC subscales will be analyzed as continuous variables using linear regression (or a suitable transformation). Course completion as a binary variable will be analyzed using logistic regression.

#### Compliance Analysis

We will report descriptively adherence to the intervention (number of training modules and coach sessions completed). Change in competency score will be reported descriptively by level of intervention received, in a dose-response type analysis.

#### Process Evaluation

Process data will be used to facilitate interpretation of the main trial results. Findings from the various data sources will be triangulated and used to develop explanatory hypotheses about potential differences in training delivery and participation across arms, subgroups of participants and coaches. We may conduct further analyses to test hypotheses generated from the integration of the process evaluation and trial outcome data (eg, on whether the duration of time taken to complete the training influences outcomes).

### Ethics Approval

Institutional review board approvals have been obtained from Sangath (the implementing organization in India; VP_2015_017); Harvard Medical School (IRB21-1197), United States (the sponsor; IRB21-1197); and the London School of Hygiene and Tropical Medicine, United Kingdom (a collaborating organization; 26565).

### Dissemination Plan

A multimodal dissemination strategy will share findings with diverse stakeholders. Strategies will include scientific publications, training manuals and videos, and web-based dissemination. All publications will be in an open access format and authorship will be determined according to the International Committee of Medical Journal Editors’ guidelines [28]. Targeted stakeholders will include practitioners, academics, NGOs, authorities involved in education and health, policy makers, and donors at state and national levels.

## Results

Participant sensitization sessions began on March 31, 2022. The first participant was enrolled on April 4, 2022, and their 6-week assessment was completed on May 18, 2022.

### Discussion 

Although India has been an incubator for some of the most widely cited task-sharing innovations in mental health care, the lack of implementation of these interventions at scale is a major challenge. The current trial will address this critical implementation gap by evaluating DT for nonspecialists, recruited from a range of contexts in India. This research expands on prior workforce development studies by evaluating 2 approaches to implementing DT, 1 self-guided and the other supported by remote individualized coaching, with a specific focus on competencies needed to treat adolescent mental health problems [8,17,29]. The evaluation design will enable a primary analysis of the effects of DT on knowledge required to deliver a brief evidence-based problem-solving intervention. A secondary analysis will compare outcomes for the DT package when delivered with and without coaching.

The coaching protocol has been designed in part to address motivational challenges highlighted in previous research on DT [30,31]. Research has recommended blended methods that combine e-learning with face-to-face or remote human support to increase engagement [11,31,32]. We have also included provision for a technical helpline, distinct from coaching, which can be used by participants in both arms of the trial.

The strengths of this trial are including participants from varied contexts across India and assessing their competency through counseling vignettes that approximate real-life situations, thus adding to their ecological validity. However, we recognize the limitation that the study does not directly assess the applicability of the acquired knowledge in counseling settings. The latter is a future research priority.

The results from the study will provide important insights into how competencies in youth-focused psychotherapies can be developed at scale outside specialist mental health settings. Related evidence will also be produced about how to motivate and support nonspecialists in settings where expert trainers and supervisors are scarce. Such evidence can be used to shape the wider dissemination of the PRIDE problem-solving intervention, and similar low-intensity psychotherapies, to address the mental health treatment gap for adolescents in India and globally.

## Abbreviations

DT: digital training
DT-C: digital training with coaching
KOPS: knowledge of problem-solving
LMS: learning management system
MUSIC: eMpowerment, usefulness, success, interest, and caring
NGO: nongovernmental organization
PRIDE: Premium for Adolescents
SMD: standardized mean difference

## Data Availability

The data sets that will be generated during this study will be available from the corresponding author on reasonable request.
